# Negative Urgency Mediates the Effect of Family Conflict on Cannabis Positive Expectancy: The Moderating Role of Anterior Cingulate Cortex

**DOI:** 10.1111/adb.70131

**Published:** 2026-02-25

**Authors:** Rabeeh Azarmehr, Cullin J. Howard, Steven M. Kogan, Charles Geier, Assaf Oshri

**Affiliations:** ^1^ Georgia Center for Developmental Science University of Georgia Athens Georgia USA; ^2^ Department of Human Development and Family Science, College of Family and Consumer Science University of Georgia Athens Georgia USA

**Keywords:** anterior cingulate cortex, cannabis positive expectancy, family conflict, negative urgency

## Abstract

Cannabis positive expectancies, favourable beliefs about cannabis effects, are a key risk factor for cannabis initiation and problematic use during adolescence. Prior research demonstrated a robust association between cannabis positive expectancies and increased use among adolescents, yet less is known about the developmental aetiology, biobehavioural mechanisms and cognitive context that contribute to these expectancies. The present study examines the intermediary role of negative urgency, a facet of impulsivity characterized by rash action under distress. Additionally, the study investigates whether anterior cingulate cortex (ACC) activation during emotional reward processing moderates this indirect effect. We conducted a longitudinal moderated mediation model with three waves of data from the Adolescent Brain and Cognitive Development (ABCD) study, analysing 6638 youths (baseline M_age_ = 10.1 years; 47.8% female). Family conflict at baseline predicted increased cannabis positive expectancies ΔT5T7 through increases in negative urgency at T5 (*β* = 0.017, *p* < 0.001, 95% CI [0.045, 0.069]). Heightened ACC activity at T5 (anticipatory large loss), including bilateral caudal and rostral regions, intensified negative urgency's impact on cannabis positive expectancy ΔT5T7: Left caudal (*β* = 0.081, *p* < 0.001, 95% CI [0.041, 0.122]), right caudal (*β* = 0.062, *p* = 0.004, 95% CI [0.020, 0.105]), right rostral (*β* = 0.041, *p* = 0.026, 95% CI [0.001, 0.081]) and left rostral (*β* = 0.052, *p* = 0.01, 95% CI [0.012, 0.092]). This study highlights how neural activity amplifies stress‐related effects on adolescent substance use expectations, suggesting emotional decision‐making as a target for prevention.

## Graphical abstract

Family conflict was associated with higher cannabis‐positive expectancies indirectly through elevated negative urgency in youth. ACC activation during anticipatory large loss (rostral and caudal) amplified this indirect pathway, indicating that heightened ACC responsivity under distress strengthens emotion‐driven impulsivity and related expectancies.

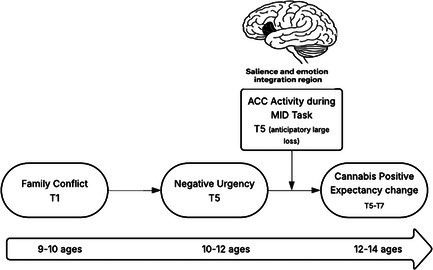

## 1 Introduction

Positive expectancies about cannabis are a key developmental risk factor for adolescent cannabis use [1]. Expectancies involve the anticipation of the benefits of cannabis use, such as relaxation or stress relief, which can contribute to attendant substance addiction vulnerability [2]. Stressful family contexts, such as family conflict, are consistently documented as a major risk factor for early initiation and continued cannabis use, partly because adolescents expect that cannabis use will help them regulate negative emotions [2]. Adverse family environmental exposures shape impulsive tendencies that underlie substance use risk [3, 4]. Accordingly, the present study focuses on negative urgency, an impulsive trait characterized by rash decision‐making in response to negative emotional states [5, 6]. Adolescents in high‐conflict families may develop more potent cannabis positive expectancies due to heightened emotional reactivity and impulsive responses to stress [7].

Individual differences in neurocircuitry linked to reward processing during adolescence can impair decision‐making by amplifying impulsive responses and increasing vulnerability to substance use behaviours [6, 8]. The anterior cingulate cortex (ACC) is a brain region that supports emotional decision‐making and extended reward processing [9]. ACC plays a key role in assessing potential rewards and evaluating losses, allowing individuals to adjust their behaviour based on situational outcomes [10]. We hypothesize that adolescents with high negative urgency may exhibit heightened ACC sensitivity to perceived losses and rewards, particularly in stressful environments. This heightened sensitivity may amplify impulsive tendencies. Using data from the Monetary Incentive Delay (MID) task [11], acquired as part of the ABCD study, this study examines whether ACC activation during rewards or losses anticipation moderates the link between negative urgency and cannabis positive expectancies. Identifying these neurobiological factors can provide a deeper understanding of the pathways connecting adolescent adversity to cannabis use risk.

### 1.1 Cannabis Positive Expectancies as a Developmental Risk Factor

Cannabis is one of the most commonly used substances, with active usage rates in adolescents between 3% and 17% worldwide [12]. A critical cognitive factor contributing to early cannabis use is the formation of cannabis positive expectancies. These expectancies refer to beliefs that using cannabis will lead to desirable outcomes such as feeling good, reducing stress or enhancing social experiences [1, 2]. Research consistently shows that adolescents who express stronger positive expectancies are more likely to initiate cannabis use, escalate their consumption over time and experience greater substance‐related problems [13]. These expectancies, once established, can lower perceived risks and heighten the appeal of cannabis, particularly during emotionally charged or stressful situations [1, 13]. Importantly, the early development of cannabis positive expectancies during adolescence significantly increases the risk for later cannabis misuse and the progression to problematic patterns of use.

### 1.2 Adolescent Development and Risk for Substance Use

During adolescence, cognitive, emotional and social processes continue to mature [14]. These developmental changes support the transition to adulthood, but can temporarily contribute to heightened emotional sensitivity, impulsivity and reward‐seeking tendencies [15]. In line with the dual systems model, still‐maturing neurobiological systems can increase adolescents' vulnerability to risky behaviour. This vulnerability may be amplified by contextual factors such as increased autonomy and shifting social environments (e.g., family dynamics [15]). Consistent with this vulnerability, epidemiological surveys indicate that rates of substance use rise sharply from mid‐ to late‐adolescence [16]. We propose that these neurodevelopmental shifts, combined with increased exposure to environmental stressors, can lead to dysregulation and impairment of decision‐making processes. This dysregulation may set the stage for substance‐related expectancies, early substance use and risk for future maladaptive trajectories. Examining these intersecting influences is critical for informing prevention strategies aimed at reducing adolescent substance use.

### 1.3 Family Conflict as a Psychosocial Risk Factor

Family conflict is a major psychosocial stressor that disrupts adolescents' emotional regulation and heightens vulnerability to substance use [17]. Children's exposure to family conflict has been conceptualized as an early form of a threatening rearing environment [18]. Extant research shows that children raised in families laden with family conflict, such as parent–child, interparental and sibling conflict, are at risk for a range of problem behaviours [19]. According to the emotional security hypothesis [20], family conflict leads to vulnerability for later psychopathology due to the development of dysregulated response patterns to threatening environments [19, 20]. Studies have shown that adolescents from high‐conflict family environments are more likely to engage in early substance use [17]. These adolescents may perceive substances as a means of escaping family‐related distress or enhancing mood, further reinforcing risky behaviours. However, despite these established associations, more research is needed to clarify the underlying mechanisms that explain how family conflict leads to the development of cannabis use positive expectancies.

### 1.4 Negative Urgency as a Mechanism Linking Family Conflict and Substance Use Risk

Negative urgency, defined as the tendency to act rashly in response to intense negative emotions, is a key individual difference that predicts vulnerability to substance use [5]. Adolescents high in negative urgency are more likely to engage in rash behaviours, including substance use, as a way to quickly reduce emotional distress [21]. Exposure to high levels of family conflict may foster the development of negative urgency by repeatedly overwhelming adolescents' emotional regulation systems and reinforcing impulsive coping patterns [22]. Research has consistently linked negative urgency to higher rates of cannabis use, alcohol misuse and other risk behaviours during adolescence [4, 23]. Although numerous studies have documented how negative urgency predicts substance use [4, 23], the developmental hypothesis that negative urgency acts as a mechanism linking family conflict to downstream cannabis positive expectancy remains to be investigated.

### 1.5 ACC and Emotional Decision‐Making

The ACC plays a central role in emotional decision‐making by monitoring emotional states, detecting conflicts and guiding behaviour under conditions of uncertainty [10, 24]. As a critical part of the brain's extended reward system, the ACC is critically involved in evaluating potential rewards and losses, helping individuals make decisions and adjust their actions based on anticipated outcomes [25]. Research suggests that heightened ACC activation during emotional reward or loss anticipation may amplify impulsive tendencies, making adolescents more prone to prioritize immediate emotional relief over long‐term consequences [26]. Given the ACC's connections to affective regulation and impulsivity [10, 24], adolescents with high negative urgency and heightened ACC activation may be particularly vulnerable to forming stronger cannabis positive expectancies as a coping strategy. Thus, ACC activation may serve as a critical potentiating neurobiological context linking emotional impulsivity to risky substance‐related beliefs in adolescents.

### 1.6 Current Study

**FIGURE 1:**
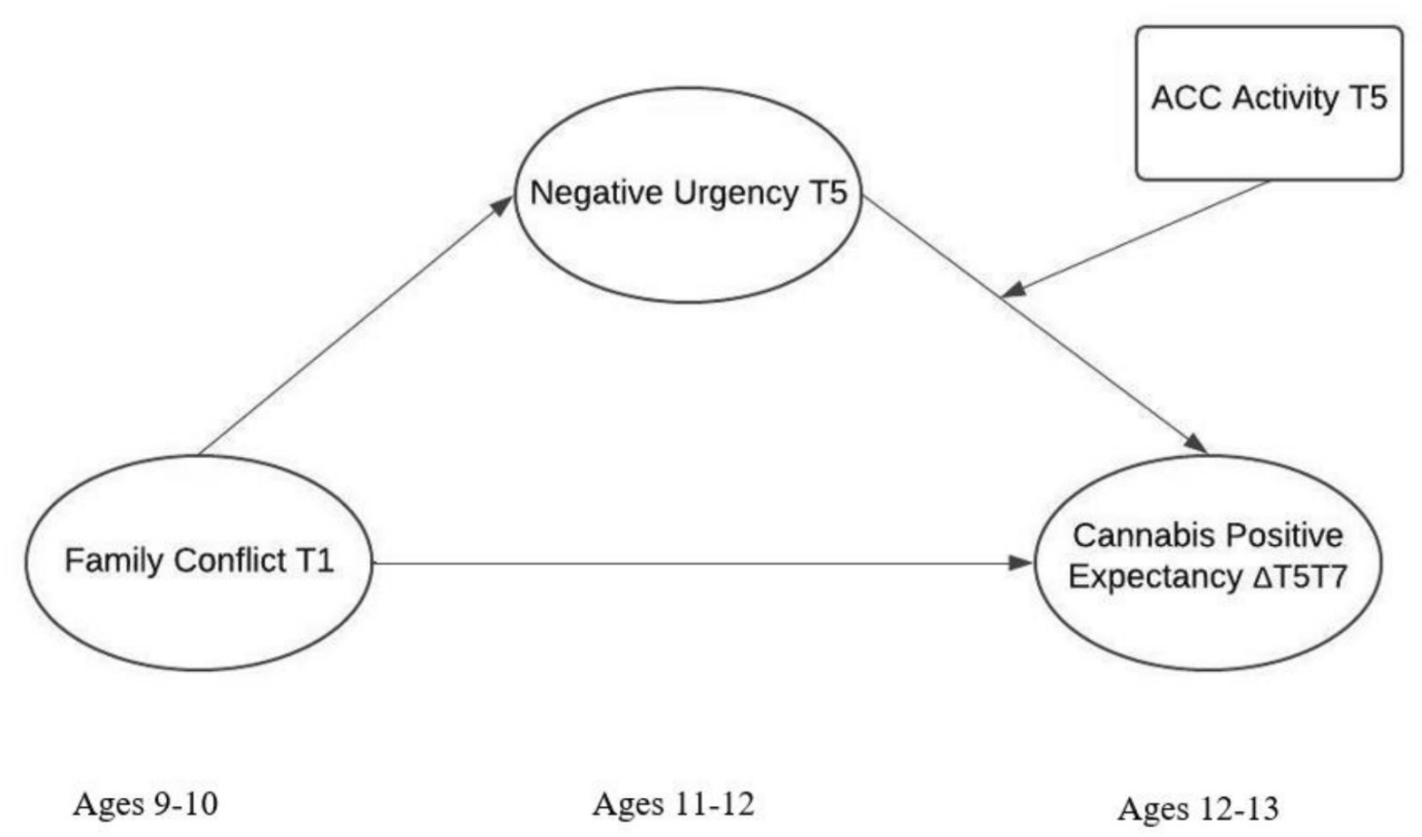
Conceptual model of study hypotheses. *Note:* ACC = anterior cingulate cortex; T1 or baseline = time‐point one; T5 = time‐point five (24 months after baseline); T7 = time‐point seven (36 months after baseline); ΔT5T7 = time‐point five to time‐point seven latent changes.

In the current study, we tested a longitudinal moderated mediation model using three waves of data from the ABCD study. Specifically, we examined two primary hypotheses: (1) negative urgency mediates the association between family conflict and cannabis‐positive expectancies, and (2) ACC activation moderates the link between negative urgency and cannabis‐positive expectancies. We hypothesized that family conflict at Time 1 would predict higher levels of negative urgency at Time 5, which in turn would be associated with increases in cannabis‐positive expectancy from T5 to Time 7. Finally, we tested whether ACC activation during the MID task moderated the indirect pathway linking family conflict to changes in cannabis‐positive expectancy via negative urgency (Figure 1). This work aims to identify emotional and neurobiological mechanisms underlying adolescent substance use risk.

## 2 Methods and Materials

### 2.1 Sample

All hypotheses were tested using data from the Adolescent Brain and Cognitive Development (ABCD) study (data release 5.0), an ongoing, nationally representative, multi‐site longitudinal study of adolescent brain development and mental health. The ABCD cohort initially recruited 11 878 youth (ages 9–10 at baseline) across 21 research sites in the United States using a school‐based stratified probability sampling framework designed to approximate national sociodemographic distributions [27]. Participants have been followed longitudinally with repeated assessments every 1–2 years, yielding strong retention rates (approximately 85%–90% across early waves) [28]. Institutional Review Boards at participating universities approved all study procedures, and adult/youth participants provided consent/assent prior to participation in the study. Full details regarding the ABCD study aims, design, recruitment and procedures can be found elsewhere [14, 29]. The data for the present analysis were accessed from the ABCD study, held in the NIH Brain Development Cohorts (NBDC) Data Sharing Platform, including the baseline (T1; M_age_ = 10.1; 47.8% female), 2‐year (T5; ages 11–12) and 3‐year follow‐up (T7; ages 12–13) assessments. The sample's racial–ethnic composition was 52.0% European American, 15.0% African American, 20.3% Latino, 2.1% Asian/Pacific Islander and 10.5% Other. Although the baseline sample consisted of 11 878 children, our final analysis utilized only those with MRI imaging data at T5, resulting in a sample of 6808 participants.

### 2.2 fMRI Data Acquisition

Neuroimaging data were obtained using Siemens, General Electric or Philips 3T scanners equipped with a 32‐channel standard adult‐size head coil. Task‐based stimuli were programmed and presented using E‐Prime Professional 2.0 Versions 2.0.10.356 or later. The full scanning protocol and task descriptions can be found in Casey and colleagues [14]. The ABCD Data Analysis and Informatics Core processed all imaging data used for this analysis using validated methods implemented in other large‐scale imaging studies [29]. Using the imaging summary variables provided in the ABCD repository, we excluded participants by following the recommended quality control protocol [29]. Complete imaging data remained for 6808 participants at T5.

### 2.3 Measures

#### 2.3.1 fMRI Task

The MID task was used to evaluate the neural activation of reward processing [11]. The MID task assesses the anticipation and outcome of rewards and losses, as well as trial‐by‐trial motivation through rapid responses aimed at winning or avoiding losses. Identical to the task described in [14], it consists of three phases: anticipation, probe and outcome (feedback). Each trial starts with a cue (lasting 2000 ms) that indicates the potential gain or loss (e.g., Win $0.20, Win $5, Lose $0.20, Lose $5 or no monetary stake). After the cue, a jittered fixation cross appears for 1500–4000 ms. This is followed by a target probe (lasting 150–500 ms), during which participants must respond quickly to win or prevent losing money. After the target, feedback is given, informing participants of their trial outcome (e.g., ‘You Win $5!’, ‘You Keep $0.20’, ‘You Lose $5’ or ‘No Money At Stake!’). The feedback duration is adjusted to be 2000 ms minus the target duration.

In the ABCD study, the MID task consists of 12 optimized trial orders, each with 50 trials (10 per trial type) in pseudorandom order. Participants complete two runs in the scanner, totaling 100 trials with 20 per trial type, each lasting 5:42 min. The task is tailored to each participant's average reaction time (RT) during a practice session, adjusting the difficulty to achieve 60% accuracy using the mean RT plus two standard deviations. This study focused on activation in bilateral caudal and rostral ACC during the anticipation phase of the large loss and large reward conditions.

#### 2.3.2 Family Conflict

The Family Environment Scale, family conflict subscale consists of nine true/false items that assess the frequency of openly expressed anger, criticism and conflict within the family (e.g., ‘We fight a lot in our family’) [30]. All items were coded in the same direction, and the factor structure was evaluated via confirmatory factor analysis (CFA) using the weighted least squares mean and variance adjusted estimator (WLSMV), which is appropriate for modelling dichotomous indicators such as the true/false items on the conflict scale. A latent factor comprising seven items was used to assess family stressors. Two items from the original nine‐item family conflict subscale were excluded (*If there's a disagreement in our family, we try hard to smooth things over and keep the peace*; *in our family, we believe you do not ever get anywhere by raising your voice*) due to poor loadings with the underlying factor (see Table 1 for items and factor loadings, *χ* ^2^(12) = 444.27(12), root mean square error of approximation [RMSEA] = 0.06, CFI/TLI = 0.96/0.93, SRMR = 0.06).

**TABLE 1:** Items factor loadings for family stress, negative urgency and cannabis positive expectancy.

Factors and indicators	Factor loadings
Family conflict T1	
We fight a lot in our family	0.80
Family members rarely become openly angry (r)	0.49
Family members sometimes get so angry they throw things	0.71
Family members hardly ever lose their tempers (r)	0.59
Family members often criticize each other	0.63
Family members sometimes hit each other	0.73
Family members often try to one‐up or outdo each other	0.51
Negative urgency T5	
When I feel bad, I will often do things I later regret in order to make myself feel better now.	0.63
Sometimes when I feel bad, I cannot seem to stop what I am doing even though it is making me feel worse.	0.66
When I feel rejected, I will often say things that I later regret.	0.54
Cannabis positive expectancy T5, T7	T5 T7
Marijuana helps me relax and feel less stressed	0.77, 0.77
Using marijuana makes me more creative	0.76, 0.79
I enjoy social gatherings more when I use marijuana	0.74, 0.80

*Note:* All factor loadings *p* < 0.01, r = reverse coded.

#### 2.3.3 Negative Urgency

The UPPS‐P Impulsive Behavior Scale–Youth Version (20 items) was used to assess impulsivity [31]. For the present study, only the four items comprising the negative urgency subscale were used (e.g., ‘When I am upset, I often act without thinking’). Items are rated on a 4‐point Likert scale ranging from 1 (*not at all like me*) to 4 (*very much like me*). A latent factor comprising the three‐item negative urgency trait was created using CFA at T5. One item (*When I am upset, I often act without thinking*) was excluded from the factor due to its inadequate factor loading, which suggested that it did not correlate well with the latent factor intended to be measured (see Table 1 for items and factor loadings). This three‐item negative urgency factor structure was just identified.

#### 2.3.4 Cannabis Positive Expectancy

Positive cannabis expectancies were assessed with the Marijuana Effect Expectancy Questionnaire–Brief (MEEQ‐brief) [32]. This measure consists of three items capturing beliefs that marijuana use produces positive or rewarding outcomes (e.g., ‘Marijuana helps a person relax and feel less tense’). Items are rated on a 5‐point Likert scale from 1 (*strongly disagree*) to 5 (*strongly agree*). All items are coded in the same direction, and the factor structure was evaluated at each time point using CFA. We then analysed the latent change in positive expectancy from T5 to T7 (ΔT5T7) by computing a multi‐indicator latent change model [33]. A positive change score indicates increases in cannabis positive expectancies from T5 to T7, whereas negative change scores signal decreases in cannabis positive expectancies over this period. The latent change model was just identified (see Table 1 for items and factor loadings).

### 2.4 Covariates

Youth biological sex (1 = female, 2 = male), age, minority status (White = 0, racial–ethnic minority = 1), parents' drug use problem at T1, scanner‐ID, mean head motion T5, state‐level legal status of cannabis use at the time of assessment and household income ranging from 1 = Less than $5.000 to 10 = $200.000 were included as covariates.

### 2.5 Analytic Plan

Hypotheses were tested using structural equation modeling (SEM) in Mplus Version 8.8 [34]. All models included propensity weights to calibrate ABCD distributions to nationally representative controls from the American Community Survey to mitigate potential selection bias in the ABCD sampling and recruitment process. To account for the nested nature of the ABCD dataset, all SEM analyses in Mplus were estimated using TYPE = COMPLEX, with CLUSTER = family_id to account for non‐independence of siblings and STRATIFICATION = scanner_serial_number to account for site/scanner effects. The criteria for evaluating model fit were as follows: a maximum value of 0.06 for the RMSEA, 0.08 for the standardized root mean squared (SRMR) and a minimum value of 0.90 for the comparative fit index (CFI) [35]. We used the WLSMV, which is robust to several kinds of non‐missing completely at random (MCAR) missingness [36] and is better suited to handle latent variables with categorical indicators (e.g., dichotomous items from family conflict subscales) [37].

We assessed the measurement model by confirming the unidimensionality of family conflict (T1), negative urgency (T5) and cannabis positive expectancy (T5 and T7) using CFA. We then calculated latent change scores for the primary outcome variable, cannabis positive expectancy, between T5 and T7 (ΔT5T7) to capture the dynamic nature of expectancy changes over time [38]. Next, we estimated the structural model by testing the direct effect of the latent factor of family conflict at T1 on negative urgency at T5 and change in cannabis positive expectancies from T5 to T7. Then, negative urgency was examined as a mechanism in the indirect link between family conflict and change in cannabis positive expectancies (ΔT5T7). The significance of the model's indirect effects was tested using bootstrapping procedures (bootstrap = 5000) [39].

Finally, we examined if the indirect effect of family conflict on changes in cannabis positive expectancies through negative urgency was moderated by ACC activity during the MID task (i.e., a conditional indirect effect). Our regions of interest (ROIs) included the bilateral caudal and rostral ACC, which were defined using the Desikan–Killiany cortical parcellation atlas [40]. This anatomically based parcellation allowed us to identify distinct subdivisions of the ACC in both hemispheres, providing a consistent framework for examining regional differences in structure and function. Latent interactions between negative urgency and different ACC regions' activity were estimated in Mplus using the XWITH function under TYPE = RANDOM; because of this specification, chi‐square–based model fit indices are not available. Significant interaction terms for this moderation were probed using simple slope analysis with moderators at ±1 SD from the mean [41], and regions of significance were identified using the Johnson–Neyman approach [42]. We applied the Benjamin–Hochberg method false discovery rate (FDR) correction to reduce the risk of Type I error [43].

## 3 Results

The correlation analysis with variables summary scores suggested that variables evinced bivariate associations in the expected directions: family conflict at T1 was associated with higher levels of negative urgency at T5 and cannabis positive expectancy at T5 and T7. Negative urgency at T5 was associated with higher levels of cannabis positive expectancy at T5 and T7 (Table 2).

**TABLE 2:** Descriptive statistics and bivariate correlations among study variables (*N* = 6808).

Variable	1	2	3	4	5	6	7	8	9	10	11	12	13	14
1. Family conflict T1	—													
2. Negative urgency T5	0.81**	—												
3. Cannabis positive expectancy T5	0.12	0.165**	—											
4. Cannabis positive expectancy T7	0.32**	0.141**	0.538**	—										
5. Left rostral ACC T5	0.33**	−0.024*	0.006	0.005	—									
6. Right rostral ACC T5	0.28**	−0.016	0.009	0.009	0.821**	—								
7. Left caudal ACC T5	0.16	−0.024	0.01	0.005	0.566**	0.574**	—							
8. Right caudal ACC T5	0.13	−0.015	0.009	0.012	0.57**	0.603**	0.845**	—						
9. Youth age T1	0.013	0.032**	0.0127**	0.017*	0.005	0.015	0.010	0.013	—					
10. Youth biological sex	−0.44**	−0.075**	−0.021	−0.002	0.01	0.002	0.04*	0.035*	−0.049**	—				
11. Youth minority status	0.10**	0.061**	0.083*	0.014	0.031	0.01*	0.003*	0.012	0.022*	0.013	—			
12. Parent drug use problems T1	0.011	0.02	0.005*	0.006*	0.022	0.012	0.002	0.008	0.003	0.005	0.014	—		
13. Cannabis law	0.050*	0.025*	−0.057*	−0.48*	0.005	0.005	−0.005	0.009	0.016	0.011	−0.099*	0.013	—	
14. Family income	−0.083*	−0.059*	0.54*	0.57*	0.040*	0.035*	0.014	0.02	0.035	0.04*	−0.01	−0.16*	−0.86*	—
Mean SD	2.52 1.95	7.71 2.33	7.13 2.92	7.67 2.99	−0.029 0.239	−0.013 0.225	0.020 0.207	0.030 0.198	9.48 0.51	1.46 0.50	1.97 1.32	0.16 0.55	2.16 0.73	7.22 2.42

*Note:* Biological sex coded as 1 = female, 2 = male; race coded as White = 0, racial–ethnic minority = 1.

Abbreviations: ACC = anterior cingulate cortex, SD = standard deviation, T1 = time‐point one, T5 = time‐point five, T7 = time‐point seven.

* Correlation is significant at the 0.05 level (2‐tailed).

** Correlation is significant at the 0.01 level (2‐tailed).

### 3.1 Direct Effects

As shown in Table 3, family conflict at T1 was positively associated with negative urgency at T5. Furthermore, negative urgency at T5 was linked to cannabis positive expectancy ΔT5T7. However, family conflict at Time 1 did not directly predict cannabis positive expectancy ΔT5T7.

**TABLE 3:** Moderated mediation model of the associations between family conflict, negative urgency, cannabis positive expectancy ΔT5T7 and ACC activity (*N* = 6808).

	*β* (SE)	*p*	*B*	Bootstrapped 95% CI
Direct effects				
Family conflict T1 ➔ negative urgency T5	0.21 (0.02)	< 0.001	0.44	[0.175, 0.263] **
Family conflict T1 ➔ cannabis positive expectancy ΔT5T7	0.007 (0.02)	0.07	0.02	[−0.038, 0.052]
Negative urgency T5 ➔ cannabis positive expectancy ΔT5T7	0.084 (0.02)	< 0.001	0.13	[0.042, 0.127] **
Indirect effects				
Family conflict T1 ➔ negative urgency T5 ➔ cannabis positive expectancy ΔT5T7	0.017 (0.002)	< 0.001	0.057	[0.045, 0.069] **
Control variables				
Youth age T1 ➔ cannabis positive expectancy ΔT5T7	0.13 (0.01)	< 0.001	0.015	[0.078, 0.118] **
Youth biological sex ➔ cannabis positive expectancy ΔT5T7	0.023 (0.01)	0.15	0.039	[−0.009, 0.055]
Youth minority status ➔ cannabis positive expectancy ΔT5T7	0.006 (0.01)	0.63	0.004	[−0.019, 0.030]
Parent drug use problems ➔ cannabis positive expectancy ΔT5T7	0.04 (0.01)	< 0.001	0.07	[0.037, 0.071] **
Cannabis law ➔ cannabis positive expectancy ΔT5T7	0.02 (0.01)	0.047	0.04	[0.001, 0.004] *
Family income ➔ cannabis positive expectancy ΔT5T7	0.003 (0.01)	0.081	0.005	[−0.028, 0.03]
Interaction effects				
Negative urgency T5 × left caudal ACC ➔ cannabis positive expectancy ΔT5T7	0.081 (0.02)	< 0.001	0.61	[0.041, 122] **
Negative urgency T5 × right caudal ACC ➔ cannabis positive expectancy ΔT5T7	0.062 (0.02)	0.004	0.48	[0.020, 0.105] **
Negative urgency T5 × left rostral ACC ➔ cannabis positive expectancy ΔT5T7	0.052 (0.02)	0.01	0.33	[0.012, 0.092] *
Negative urgency T5 × right rostral ACC ➔ cannabis positive expectancy ΔT5T7	0.041 (0.02)	0.026	0.028	[0.001, 0.081] *

*Note:* Sex coded as 1 = female, 2 = male; model fit was good: *χ* ^2^(220) = 1572.01, *p* = < 0.01; RMSEA = 0.03; CFI = 0.92; SRMR = 0.03.

Abbreviations: *B* = unstandardized beta coefficient, CI = confidence interval, SE = standard error, T1 = time‐point one, T5 = time‐point five, T7 = time‐point seven, *β* = standardized beta coefficient.

* *p* < 0.05.

** *p* < 0.01.

### 3.2 Mediation

We tested the associations between family conflict T1 and cannabis positive expectancy ΔT5T7 via negative urgency at T5. The mediation analysis model fit was good: *χ* ^2^(220) = 1572.01, *p* = < 0.01; RMSEA = 0.03; CFI = 0.92; SRMR = 0.03, and indicated that family conflict was linked to increases in cannabis positive expectancies 3 years later through increases in negative urgency (Table 3).

### 3.3 Moderated Mediation

**FIGURE 2:**
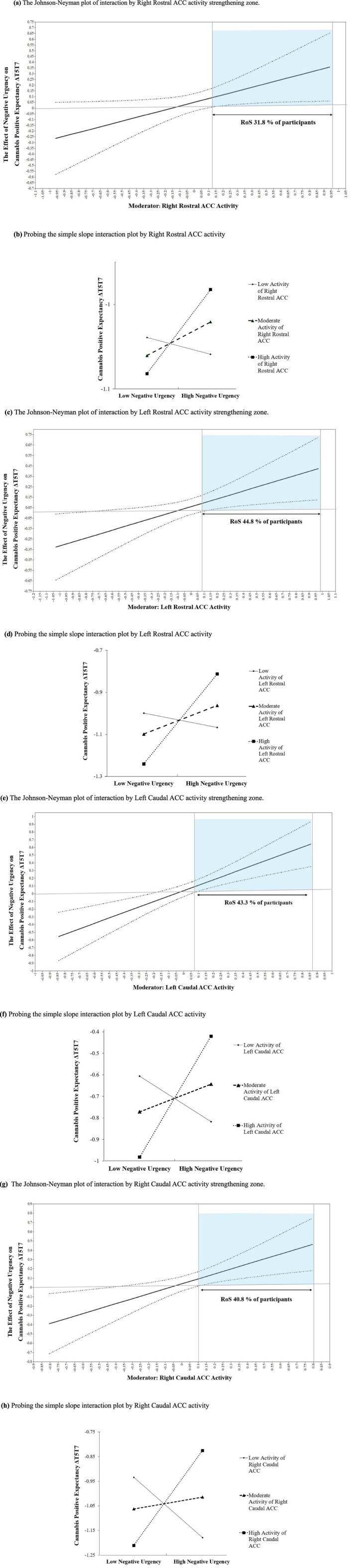
(a) The Johnson–Neyman plot of interaction by right rostral ACC activity strengthening zone. (b) Probing the simple slope interaction plot by right rostral ACC activity. *Note:* Interpretation of the moderating effect of ACC on the effect of negative urgency on youth cannabis positive expectancy. The upper panel presents the Johnson–Neyman plot, and the lower panel presents simple slope interpretation. In the upper panel, the shadowed area indicates regions of significance. The *x*‐axis represents the ACC (right rostral) activity at T5. The *y*‐axis represents the unstandardized coefficient of the effects of negative urgency on youth cannabis‐positive expectancy ΔT5T7. The solid line indicates the main effect, and the two light lines suggest 95% of the CI of the main effect. Regions where both upper and lower 95% CI lines are both above 0 (i.e., shaded area) are RoS for the moderating effect. According to the right‐hand RoS, as ACC activity (right rostral) increased, those with high ACC activity (right rostral) showed an increasingly positive association between high negative urgency and youth cannabis‐positive expectancy ΔT5T7. Frequency analysis on the moderator (right rostral ACC) showed that 31.8% of participants fell within the RoS. The lower panel presents the simple slope interpretation of this moderating effect. (c) The Johnson–Neyman plot of interaction by left rostral ACC activity strengthening zone. (d) Probing the simple slope interaction plot by left rostral ACC activity. *Note:* Interpretation of the moderating effect of ACC on the effect of negative urgency on youth cannabis positive expectancy ΔT5T7. The upper panel presents the Johnson–Neyman plot, and the lower panel presents simple slope interpretation. In the upper panel, the shadowed area indicates regions of significance. The *x*‐axis represents the ACC (left rostral) activity at T5. The *y*‐axis represents the unstandardized coefficient of the effects of negative urgency on youth cannabis‐positive expectancy ΔT5T7. The solid line indicates the main effect, and the two light lines suggest 95% of the CI of the main effect. Regions where both upper and lower 95% CI lines are both above 0 (i.e., shaded area) are RoS for the moderating effect. According to the right‐hand RoS, as ACC activity (left rostral) increased, those with high ACC activity (left rostral) showed an increasingly positive association between high negative urgency and youth cannabis‐positive expectancy ΔT5T7. Frequency analysis on the moderator (left rostral ACC) showed that 44.8% of participants fell within the RoS. The lower panel presents the simple slope interpretation of this moderating effect. (e) The Johnson–Neyman plot of interaction by left caudal ACC activity strengthening zone. (f) Probing the simple slope interaction plot by left caudal ACC activity. *Note:* Interpretation of the moderating effect of ACC on the effect of negative urgency on youth cannabis positive expectancy ΔT5T7. The upper panel presents the Johnson–Neyman plot, and the lower panel presents a simple slope interpretation. In the upper panel, the shadowed area indicates regions of significance. The *x*‐axis represents the ACC (left caudal) activity at T5. The *y*‐axis represents the unstandardized coefficient of the effects of negative urgency on youth cannabis positive expectancy ΔT5T7. The solid line indicates the main effect, and the two light lines suggest 95% of the CI of the main effect. Regions where both upper and lower 95% CI lines are both above 0 (i.e., shaded area) are RoS for the moderating effect. According to the right‐hand RoS, as ACC activity (left caudal) increased, those with high ACC activity (left caudal) showed an increasingly positive association between high negative urgency and youth cannabis‐positive expectancy ΔT5T7. Frequency analysis on the moderator (left caudal ACC) showed that 43.3% of participants fell within the RoS. The lower panel presents the simple slope interpretation of this moderating effect. (g) The Johnson–Neyman plot of interaction by right caudal ACC activity strengthening zone. (h) Probing the simple slope interaction plot by right caudal ACC activity. *Note:* Interpretation of the moderating effect of ACC on the effect of negative urgency on youth cannabis positive expectancy ΔT5T7. The upper panel presents the Johnson–Neyman plot, and the lower panel presents a simple slope interpretation. In the upper panel, the shadowed area indicates regions of significance. The *x*‐axis represents the ACC (right caudal) activity at T5. The *y*‐axis represents the unstandardized coefficient of the effects of negative urgency on youth cannabis positive expectancy ΔT5T7. The solid line indicates the main effect, and the two light lines suggest 95% of the CI of the main effect. Regions where both upper and lower 95% CI lines are both above 0 (i.e., shaded area) are RoS for the moderating effect. According to the right‐hand RoS, as ACC activity (right caudal) increased, those with high ACC activity (right caudal) showed an increasingly positive association between high negative urgency and youth cannabis‐positive expectancy ΔT5T7. Frequency analysis on the moderator (right caudal ACC) showed that 40.8% of participants fell within the RoS. The lower panel presents the simple slope interpretation of this moderating effect.

All four ACC ROIs (bilateral caudal/rostral ACC) significantly moderated the effect of increased negative urgency on cannabis positive expectancy ΔT5T7 during the anticipatory large loss phase. Likewise, the conditional indirect effects (i.e., the moderation effect on the overall mediation model) for these regions were also significant, and estimates of the conditional indirect effect were calculated using Mplus code for the moderated mediation model (Table 4 [44]). Johnson–Neyman technique (Figure 2a,c,e,g [42]) and simple slope method (Figure 2b,d,f,h [41]) were used to probe the moderating role of ACC activity (e.g., right rostral and left caudal ACC) at T5 on the association between increased negative urgency at T5 and youth cannabis positive expectancy ΔT5T7. Accordingly, for participants with high levels of ACC activity, higher negative urgency was associated with higher levels of cannabis positive expectancy ΔT5T7. These results suggested that the indirect effect of family conflict on youth cannabis positive expectancy ΔT5T7 via higher levels of negative urgency was significant and positive among participants with high levels of ACC activity in all regions (left caudal = 43.3%, right caudal = 40.8%, left rostral = 44.8% and right rostral = 31.8%). Using the Benjamini–Hochberg method [43] to control the FDR across the four ACC interaction tests, the adjusted significance threshold was *p* < 0.03. All four interactions remained significant under this criterion. For youth with lower levels of ACC activity (bilateral caudal/rostral), the association between high negative urgency and cannabis positive expectancy ΔT5T7 was not significant.

**TABLE 4:** Conditional indirect effect of the associations between family conflict, negative urgency and cannabis positive expectancy ΔT5T7 with different levels of ACC activity (*N* = 6808).

Moderator levels	*B* (SE)	*β*	*p*	Bootstrapped 95% CI
Right rostral ACC T5				
−1 SD below the M (low)	−0.002 (0.003)	−0.003	0.64	[−0.008, 0.005]
M (moderate)	0.003 (0.002)	0.005	0.2	[0.002, 0.008]
+1 SD above the M (high)	0.007 (0.003)	0.01	0.01	[0.001, 0.014] *
Left rostral ACC T5				
−1 SD below the M (low)	−0.002 (0.003)	−0.003	0.49	[−0.009, 0.004]
M (moderate)	0.004 (0.002)	0.007	0.12	[−0.001, 0.008]
+1 SD above the M (high)	0.009 (0.003)	0.01	0.007	[0.002, 0.015] **
Right caudal ACC T5				
−1 SD below the M (low)	−0.003 (0.003)	−0.005	0.40	[−0.01, 0.004]
M (moderate)	0.004 (0.002)	0.007	0.13	[−0.001, 0.008]
+1 SD above the M (high)	0.010 (0.003)	0.001	0.003	[0.003, 0.016] **
Left caudal ACC T5				
−1 SD below the M (low)	−0.005 (0.003)	−0.008	0.11	[−0.012, 0.001]
M (moderate)	0.003 (0.002)	0.005	0.10	[−0.001, 0.008]
+1 SD above the M (high)	0.012 (0.002)	0.02	< 0.001	[0.005, 0.019] **

*Note:* Mean value of ACC activity was used for moderate, and one standard deviation above or below the mean was used for the high and low values, respectively.

Abbreviations: ACC = anterior cingulate cortex, *B* = unstandardized beta coefficient, CI = confidence interval, M = mean, SD = standard deviation, SE = standard error, T5 = time‐point five.

* *p* < 0.05.

** *p* < 0.01.

## 4 Discussion

Substance use risk peaks in late adolescence, and early drug use is a well‐established risk factor for later addiction. This study aimed to investigate the developmental neurocognitive aetiology of expectancy during adolescence, a robust cognitive risk factor for addiction. Prior studies have emphasized that adolescents who experience family conflict are particularly vulnerable to developing unhealthy coping strategies, including a tendency toward outcome‐positive expectancy [45]. However, the pathways connecting family conflict to substance use expectancy, particularly through interactions between behavioural responses and neural processes, are not fully understood. This study examined the role of negative urgency in mediating the association between family conflict and cannabis positive expectancy in adolescents. We also aimed to test the moderating role of ACC, a key brain region involved in emotional decision‐making, in this association. Our findings suggest that negative urgency at T5 links family conflict at T1 to increased cannabis positive expectancy ΔT5T7. Variations in ACC activity at T5 may further heighten the likelihood of drug use expectancies.

Our main goal was to investigate whether family conflict is linked to the development of cannabis positive expectations later in life. We found that exposure to family conflict does not directly lead to the development of cannabis positive expectancies in adolescents. This is consistent with empirical data, suggesting that some genetic and environmental factors might be underlying this mechanism [46]. Similarly, other research has demonstrated that childhood stressors are associated with cannabis use onset through emotional dysregulation [7]. Furthermore, another study indicated that young adults who report cannabis use had more psychological vulnerabilities, including childhood adversity and stress [47]. These findings underscore the importance of considering underlying psychological processes when examining the long‐term impact of early family conflict on substance‐related cognition.

Our findings supported the second hypothesis that negative urgency mediated the association between family conflict and cannabis positive expectancy. According to life history theory [48], individuals exposed to threatening environments during their childhood are more likely to adopt faster life history strategies, which include increased impulsivity and impulsive behaviours later in life. Our result aligns with previous research showing that family conflict is associated with increased impulsivity and emotional dysregulation, particularly among adolescents who have experienced adverse childhood events [4, 49]. According to urgency theory, negative urgency is a key predictor of the initiation of substance use and other addictive behaviours, as it often responds to intense negative emotions rather than positive ones [5]. This suggests that adolescents exposed to early family conflict may develop maladaptive coping mechanisms, where impulsive responses to negative emotions heighten the appeal of substance use [5, 49]. However, negative urgency is not the only factor; neural activity also plays a key role, making it another promising target for intervention in mitigating the effects of family conflict on cannabis expectancy in adolescents.

Lastly, we found that elevated neural activity in bilateral rostral and caudal ACC during anticipatory large loss exacerbated the indirect effect of family conflict on cannabis positive expectancy via increased negative urgency in youth. Although these subregions are functionally distinct, with rostral ACC primarily implicated in affective regulation and processing emotionally salient stimuli [50] and caudal ACC in cognitive control and adaptive decision‐making [24]. Elevated activity across both subregions suggests a shared role in emotion–cognition integration under distress.

Consistent with this interpretation, moderation effects were highly consistent across ACC subregions, as Johnson–Neyman [42] and simple slope [41] analyses yielded nearly identical patterns, with only minor differences in effect size. Such convergence likely reflects the high intercorrelation among ACC subregions during anticipatory large loss and their overlapping contribution to emotion–cognition integration, rather than discrete subregional specificity. Notably, prior work indicates that hyperactivation under distress may reflect compensatory regulation rather than effective regulation [51], highlighting that increased ACC activity does not necessarily indicate adaptive functioning or protection.

We found no significant result for anticipatory large reward. This finding aligns with loss aversion theory [52], which suggests that individuals are more sensitive to potential losses than equivalent rewards. Adolescence is marked by heightened emotional reactivity and sensitivity to negative outcomes, particularly in stressful and/or uncertain contexts [53]. For youth high in negative urgency, anticipated losses may intensify negative affect and increase reliance on maladaptive coping strategies [5, 54]. In this context, cannabis positive expectancies may function less as reward‐driven beliefs and more as emotion‐regulation expectancies aimed at alleviating distress. Thus, loss anticipation may represent a particularly salient neurocognitive context in which impulsive tendencies translate into substance‐related risk beliefs [10].

These findings have important implications for prevention and early intervention. At the family level, interventions aimed at reducing chronic conflict and improving emotional communication may help attenuate upstream environmental risk. At the individual level, prevention programmes that target distress‐driven impulsivity, such as emotion regulation or coping skills training, may be particularly beneficial for adolescents high in negative urgency. At the neurocognitive level, interventions that strengthen adaptive decision‐making under stress may further disrupt pathways linking adversity to substance‐related expectancies. Importantly, these strategies may be most effective when implemented early in adolescence.

### 4.1 Strengths and Limitations, and Conclusion

This study has several strengths, including the use of a large, nationwide longitudinal sample from the ABCD study, which enhances the generalizability of our findings. The multi‐wave data collection allowed for examining developmental changes and causal relationships over time. Additionally, the integration of self‐reported measures with fMRI data provided a comprehensive view of the behavioural and neural mechanisms underlying the link between family conflict, negative urgency and cannabis positive expectancies. However, several limitations should be noted. Key constructs were assessed using self‐report, which may introduce reporting bias and shared method variance and constrain mechanistic interpretation. Future studies incorporating multi‐informant or behavioural measures may help clarify underlying processes. Although the study's longitudinal design is a strength, it does not fully establish causality. Unmeasured confounds such as genetic liability, peer influence and socioeconomic adversity may shape observed associations. Genetically informed and quasi‐experimental designs will be important for clarifying these pathways. The fMRI data were collected during a specific task, which may not capture all relevant neural processes.

Also, the effect sizes observed in the mediation and moderation pathways were small. This pattern is consistent with findings from other ABCD‐based analyses [55, 56], where effect sizes tend to be modest due to the study's very large, diverse and representative sample and the distal nature of developmental risk processes. In such contexts, psychosocial and neurobiological factors are expected to contribute to risk across development rather than exert large isolated effects. Nevertheless, even small but reliable effects can be meaningful at the population level and inform prevention efforts by identifying early developmental pathways to substance‐related risk. Taken together, these findings highlight how family conflict and affective impulsivity jointly shape cannabis‐related expectancies during adolescence, with ACC activation further moderating this pathway. By integrating psychosocial and neurobiological perspectives, this study advances understanding of early developmental mechanisms underlying vulnerability to substance use and informs prevention efforts targeting youth exposed to family stress.

## Author Contributions

Rabeeh Azarmehr conceptualized the study, conducted the analyses, and drafted the manuscript. Assaf Oshri and Charles Geier contributed to study conceptualization and provided substantive feedback and editing on the manuscript. Cullin Howard and Steven Kogan reviewed the manuscript and contributed to editing and revisions.

## Funding

This work was supported by National Institutes of Health and National Institute on Drug Abuse, awarded to Dr. Assaf Oshri (R01 DA058334).

## Disclosure

All authors in this manuscript report no financial interests or potential conflicts of interest.

## Ethics Statement

Institutional Review Boards at participating universities approved all study procedures.

## Conflicts of Interest

The authors declare no conflicts of interest.

## Data Availability

The data supporting the findings of this study, ‘ABCD study’, are available nationwide. The data can be accessed by authorized researchers and institutions.
